# The Book World of Medicine and Science

**Published:** 1901-12-07

**Authors:** 


### THE BOOK WORLD OF MEDICINE AND SCIENCE.

First Aid Diagrams for the Use of Lecturers. (Bristol: John Wright and Co. 1901. Price for the set 27s. 6d. net, but the sheets can be obtained separately.)

Every lecturer is not a draughtsman, and the lack of suitable diagrams is often found to hamper a man greatly in his efforts to convey his meaning to his class. These diagrams are intended to supply this want so far as "first aid" lectures are concerned. On 16 large sheets over 200 diagrams are clearly printed. They are well selected for their purpose, and for small classes will, we are sure, prove very useful. Four sheets are devoted to anatomy and physiology; three to the triangular bandage, which seems to retain its popularity as a first aid appliance; two each to the roller bandage, to hæmorrhage and wounds, and to dislocations and fractures; while to artificial respiration and transport three sheets are devoted. Thus the lecturer has at hand a very large selection of diagrams, to which he may return again and again in the course of his lecture, a thing he cannot well do when he depends on blackboard illustration. From the way in which the sheets are mounted, hanging down from a bar across the top by which they are all held together, they can not only be easily displayed but also easily rolled up for removal from place to place. Practical experience before large audiences soon teaches most lecturers that few diagrams are large enough to be visible from the far end of the room, and these diagrams share in this common defect, but for small classes, such, for example, as probationers' classes at hospitals, these clearly printed illustrations will answer every purpose, and certainly will save the lecturer much trouble.

The Criminal. By Havelock Ellis. Third Edition, revised and enlarged. With 40 Illustrations. (London: Walter Scott. 1901. Price 6s.)

To describe the criminal, to give him his place in the social fabric, and to suggest how he is to be eliminated is a great task, and although it can hardly be said that the author of this work has solved the problem, he has collected a large amount of information bearing on the subject. The various antecedents which may be spoken of as causes of crime may be classed as cosmic, biological, and social, and it is chiefly with the biological factor that this book deals. It is impossible to overestimate the importance of the social factor in the production of crime. Perhaps, indeed, it may be true in the words of Lacassagne that "every society has the criminals that it deserves." But we cannot deal with this factor, nor can we estimate the vast importance of social influence in the production or prevention of crime unless we know something of the criminal himself, of his anatomical, physiological and psychological nature; and it is to this subject that this book is to a large extent devoted. The question to be answered is, What is a criminal? Is he a normal person who has wilfully broken the law? Is he the victim of a disease, such as some form of epilepsy? Is he an atavistic re-appearance of the savage in modern society? Or is he what is termed a degenerate? These are some of the questions which the criminal anthropologist has to answer, and the author of this book, in his endeavours so to do, fully sets forth the evidence which goes to show that there is a real relationship, although not an identity, between a very large and characteristic group of criminals and those congenitally abnormal groups which we term imbecile and feeble-minded. The criminal, in some of his most characteristic manifestations, is, according to this view, a congenitally weak-minded person whose abnormality, while by no means leaving the mental aptitudes unimpaired, chiefly affects the feelings and volition, so influencing conduct, and rendering him an anti-social element in society. Having, then, got hold of such an anti-social person, what are we to do with him? Clearly it is no use sending him to prison for a fortnight. To deal scientifically with the criminal "we need the absolute abolition of the fixed term of imprisonment, an absolutely indeterminate sentence." We must keep him till he is cured. The subject is treated in a thorough-going spirit, and the author does not hesitate to use whatever words seem best to explain his meaning, so that it is not exactly the book one would choose to read aloud in the drawing-room.

Jerusalem: A Practical Guide to Jerusalem and its Environs, with Excursions, etc. By E. A. Reynolds-Ball, B.A., F.R.G.S. With plan, maps, and illustrations. (London: Adam and Charles Black. 1901. Price 2s. 6d.)

Jerusalem by steamer and by train; its banks, its cafés, and its cabs. It all sounds incongruous, and the more the resources of western civilisation intrude themselves within the holy city, the more strange and out of place will they always appear to those who have thought of Jerusalem only as the birthplace of our religion, and as the spot on which the sacred events of gospel history took place. Yet for a thousand years Jerusalem, if not the resort of tourists, for tourists were not, has been the goal of travellers and pilgrims from every part of the world to which the Christian religion has spread. One must not then be surprised to find it described as the most cosmopolitan city in the world. No doubt a good deal of the romance of travel is rubbed off by reading such a guide-book as this, as indeed it is by traval itself. Nevertheless it may be satisfactory to the adventurous to know that, although Jerusalem is safe enough, there are places in the neighbourhood where one must be careful not to rouse the fanaticism of the Moslem. Even in Jerusalem itself, as Mr. Reynolds-Ball is careful to tell us, one need not dress for dinner at the hotels. So there is still something left us of the East! As a guide-book this little work is good. It will go in one's pocket; it has maps; it tells us just what to see and how to go about it; and although it enters into some discussion as to the "holy sites controversy," it is not stuffed out with history and other matters which, essential as they are for study and for evening reading, one does not want to carry with one all the day. The information given is very modern, and we can imagine that many people who have no hope of ever visiting Jerusalem may be glad to read the account given in its pages of the present state of affairs in regard to the places of pilgrimage which are therein described. It is crammed full of information.

Diet and Food Considered in Relation to Strength and Power of Endurance, Training and Athletics. By Alexander Haig, M.A., M.D.Oxon., F.R.C.P. Third edition. (London: J. and A. Churchill. 1901. Price 2s.

All depends on point of view, and if we regard questions of diet from the point of view taken by Dr. Haig in reference to uric acid and its allies, then this book is altogether admirable. Dr. Haig writes with great force, and one can quite understand the strong sense of duty by which he seems to be impelled when we find him expressing his belief that 75 per cent. of the sorrow and suffering which he as a physician is asked to relieve could have been prevented by excluding from our diet the poisons which we, most of us at least, so habitually consume. Whether we agree with it or not the book is the outcome of much careful study and is well worth reading.

